# Temporomandibular Joints’ Morphology and Osteoarthritic Changes in Cone-Beam Computed Tomography Images in Patients with and without Reciprocal Clicking—A Case Control Study

**DOI:** 10.3390/ijerph17103428

**Published:** 2020-05-14

**Authors:** Marcin Derwich, Maria Mitus-Kenig, Elzbieta Pawlowska

**Affiliations:** 1 Department of Orthodontics, Medical University of Lodz, 90-419 Lodz, Poland; elzbieta.pawlowska@umed.lodz.pl; 2 Department of Prophylaxis and Experimental Dentistry, Jagiellonian University in Krakow, 31-008 Krakow, Poland; maria.mitus@interia.pl

**Keywords:** temporomandibular joint, reciprocal clicking, osteoarthritic changes, cone-beam computed tomography

## Abstract

*Background:* Patients referred for orthodontic treatment often present symptoms of temporomandibular joints’ disorders (TMD), predominantly clicking. The objective was to analyze the morphology of the temporomandibular joints in cone-beam computed tomography (CBCT) images based on the presence of reciprocal clicking before orthodontic treatment. *Methods:* 105 participants took part in the study. 210 temporomandibular joints (TMJs) were allocated into one of two groups regarding the presence of reciprocal clicking. Morphology of condyle’s head, glenoid fossa, and articular eminence as well as condylar head position in the glenoid fossa and osteoarthritic changes in the area of the condylar head were examined for each TMJ in the CBCT images. Statistical analysis was performed with STATISTICA version 12.0. The following tests were performed: U-Mann Whitney, Kruskal-Wallis, t-Student, and chi-square. The statistical significance level was *p* = 0.05 for all the measurements included. *Results:* Significantly smaller condylar A-P dimension (*p* = 0.040) characterized temporomandibular joints with reciprocal clicking. Condyles were substantially more often positioned posteriorly (*p* = 0.043) and were significantly more often accompanied by subcortical cysts and pathologic osteoarthritic bone changes (*p* < 0.001). *Conclusions:* The early stages of internal derangements stay with alterations in morphology and position of TMJs as well as with the presence of osteoarthritic changes.

## 1. Introduction

The anatomical structures forming the temporomandibular joint include: the articular surface on the temporal bone (which consists of the articular fossa, called the mandibular fossa, that is localized in the posterior part and the anteriorly localized articular eminence), the condyle, the articular disc, the bilaminar zone (also known as the retro-discal tissue), and the joint capsule strengthened with ligaments [1,2,3]. There are four differently described types of condyle’s shape in the projection in the frontal plane: convex, flat, angular, and round, among which the most common type is convex (58%) [4].

The articular disc is of biconcave shape with a thicker anterior and posterior border when compared to the intermediate zone [1,3]. The articular disc is attached to the condyle by the discal collateral ligaments (both the lateral and the medial). Therefore, pure rotation is the only type of movement that can occur within the condyle-disc complex [1]. The maximum extent of described rotational movement depends on the length of the discal ligaments and the retro-discal lamina, the anterior capsular ligaments, morphology of the disc, degree of interarticular pressure, and superior lateral pterygoid muscle activity [1].

While opening the mouth, the condyle moves forward to the top of the articular eminence. In healthy joints, the position of the articular disc is easily self-positioned above the condyle during the entire opening and closing movements, not only because of the presence of the collateral ligaments but also because of the biconcave morphology of the disc and simultaneous interarticular pressure provided by elevator muscles [1].

Ligaments cannot be stretched since they have no elasticity. Once elongated, they do not return to their original length. When the ligaments become elongated, the superior lateral pterygoid muscle hypertonicity may induce alteration of the articular disc morphology. Because the superior oblique pterygoid muscle pulls the disc into the forward and medial direction, the posterior band of the disk may become thinner. Therefore, the articular disc loses its ability to self-position above the condyle and may become displaced [1]. 

There are four clinical stages of anterior disc displacement [5]: stage I—anterior disc displacement with reduction, stage II—anterior disc displacement with reduction with intermittent locking, stage III—anterior disc displacement without reduction, and stage IV—anterior disc displacement without reduction with perforation of the disc or posterior attachment tissues.

Anterior disc displacement with reduction is considered to be the most common type of internal derangements of the temporomandibular joints [6,7]. The articular disc is positioned anteriorly to the condyle. When the mandible opens, the condyle moves across the posterior part of the disk. Then, it moves with the disk to the top of the articular eminence. While closing the mouth, the disk moves back with the condyle to the glenoid fossa. Because of simultaneous hyperactivity of superior lateral pterygoid muscle, alteration in disc morphology, and ligaments elongation, the disc slips back anteriorly. Anterior disc displacement with reduction is characterized by reciprocal clicking, which appears twice during mouth opening and closing when the condyle moves across the posterior border of the disk [1].

The study aimed to compare the morphology of the temporomandibular joints, including mandibular condyle, glenoid fossa, articular eminence, and the position of the condyle in glenoid fossa and presence of osteoarthritic changes in cone-beam computed tomography (CBCT) images based on the presence of reciprocal clicking before orthodontic treatment.

## 2. Materials and Methods 

### 2.1. Participants 

**Figure 1:**
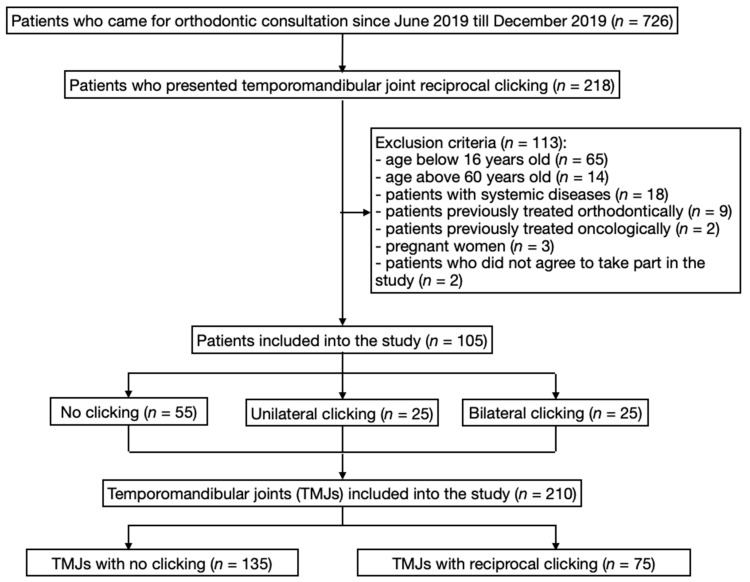
The flow chart of participation.

For this research we examined the same group of patients, which we had already presented in our previous research [8]. There were 105 patients included into the study (79 women and 26 men, mean age: 24.93±7.74 years). All of these patients had been referred for the specialist orthodontic consultation. We examined 210 temporomandibular joints. Inclusive criteria: the age between 16 and 60 years old, people who wanted to participate in the study, with no systemic diseases, and those who had never been treated orthodontically. Excluded from the study were all the cases with: the age below 16 and above 60 years old, anterior disc displacement without reduction, temporomandibular joint ankylosis, pregnancy, rheumatological diseases, oncological diseases, people who had undergone radiotherapy (especially in the area of head and neck), patients who had ever had any traumas in the field of head and neck, people who had been treated orthodontically at least once in the past, and those who did not agree to take part in the study [1,8,9]. All patients received and signed informed consent. The study was approved by the Independent Bioethics Committee for Scientific Research (RNN/74/20/KE) and was conducted with the ethical principles of the World Medical Association Declaration of Helsinki. Figure 1 presents the flow chart of participation.

### 2.2. Protocol 

Each patient underwent standard pre-orthodontic-treatment examination, which was extended by taking additional cone-beam computed tomography (CBCT) images of the temporomandibular joints (TMJs) [10]. The indications for taking additional CBCT scans were: either the presence of the TMJs’ reciprocal clicking or asymptomatic patients who presented at least one of the below mentioned manifestations: occlusal instability, teeth attrition, masticatory muscles hypertension, teeth clenching, and family history of temporomandibular joint disorder [1,8,9].

The patients were allocated into the groups based on the presence of reciprocal clicking: control with no clicking in both joints, the group with reciprocal clicking in only one temporomandibular joint, and group with reciprocal clicking in both temporomandibular joints. Lastly, the groups were subdivided into two groups: Group A (control group) – temporomandibular joints without reciprocal clicking and Group B – temporomandibular joints with reciprocal clicking.

### 2.3. Imaging Procedures 

Cone-beam computed tomography (CBCT) imaging was conducted on MyRay Hyperion X9 3D (company: CEFLA, Imola, Italy). The parameters of exposition were 90kV, 18mAs, and exposition time of 3.6 s. Established field of view (FOV) was 8 cm × 5 cm, and the thickness of slices was 0.3 mm [8].

### 2.4. Measurements 

The 0.3-mm thickness axial view of the condyle in which the condyle had the maximum mediolateral dimension was selected for further measurements. The sagittal axis was established as a line, which was perpendicular to and, at the same time, crossing the middle of the line connecting the mesial and distal end of the condyle. The obtained sagittal and coronal views were further examined and measured. All the measurements were performed with the use of iRYS Software version 6.2 (company: CEFLA, Imola, Italy) [8]. 

#### 2.4.1. Morphology of the Mandibular Condyle 

According to Yale’s classification based on condyle’s superior surface view, each condyle head was classified as one of four types: convex, flattened, angled, and rounded [4]. The shape of the condyle head was assessed in the obtained coronal view [8]. 

The condylar width was a maximum mediolateral width measured in the axial view [8]. 

**Figure 2:**
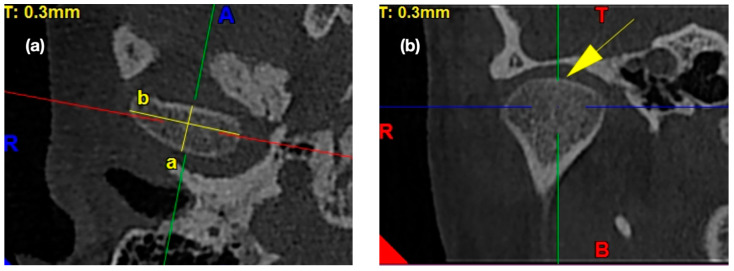
The exemplary lines, points, and angles in the TMJ CBCT scans used for measurements regarding the morphology of the mandibular condyle. (**a**) Morphology of mandibular condyle in the axial view: /a/ condylar A-P dimension, /b/ condylar width. (**b**) Shape of condyle head in the coronal view: condyle’s superior surface view assessment.

The condylar A-P dimension was measured from the most anterior to the most posterior point on the condylar head as a perpendicular distance to the maximum mediolateral width, crossing it in the middle [8]. Figure 2 presents the exemplary lines, points, and angles in the TMJ CBCT scans used for measurements regarding the morphology of the mandibular condyle.

#### 2.4.2. Morphology of the Glenoid Fossa (Mandibular Fossa, Articular Fossa)

The shape of glenoid fossa was assessed in the obtained sagittal views [8]. Classification of shapes of the fossae included: oval, triangular, angled, trapezoidal, and other types [11].

Glenoid fossa depth was measured as a perpendicular distance from the highest point of the glenoid fossa to the fossa basal line in the obtained sagittal view [8].

Fossa basal line was traced from the lowest point of the articular eminence to the lowest point of external auditory meatus in the obtained sagittal view [8].

Glenoid fossa length was measured from the lowest point of the articular eminence to the anterior part of the tympanic part of the temporal bone along the basal line, connecting the lowest point of the articular eminence with the lowest point of the external auditory meatus [8].

The glenoid fossa divergence angle was the angle measured between two lines known as the PE (posterior eminence line) and AT (anterior tympanic line) in the obtained sagittal views [8].

The PE line (posterior eminence line) was traced as the best fitting line, which was tangent to the posterior wall of the articular eminence [8].

The AT line (anterior tympanic line) was traced as the best fitting line, which was tangent to the anterior wall of the tympanic part of the temporal bone [8].

**Figure 3:**
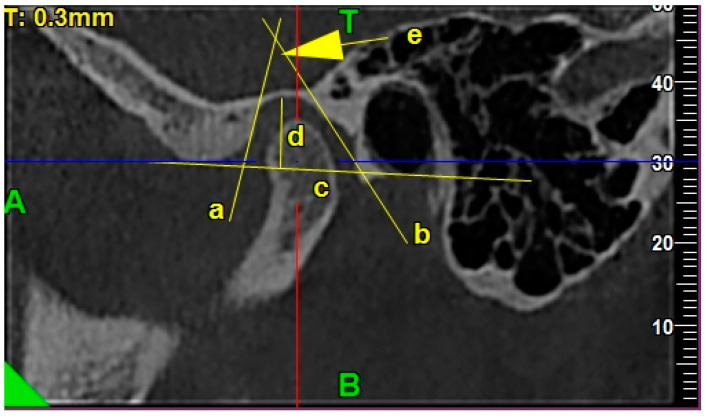
The exemplary lines, points, and angles in the TMJ CBCT scans used for measurements regarding the morphology of the glenoid fossa in the sagittal view: /a/ PE line, /b/ AT line, /c/ glenoid fossa basal line, /d/ glenoid fossa depth, and /e/ glenoid fossa divergence angle.

Figure 3 presents the exemplary lines, points, and angles in the TMJ CBCT scans used for measurements regarding the morphology of the glenoid fossa in the sagittal view.

#### 2.4.3. Morphology of the Articular Eminence

Articular eminence height was measured as a perpendicular distance from the lowest point on an articular eminence to the eminence basal line, measured in the obtained sagittal view [8].

The eminence basal line was traced from the highest point of articular fossa and was tangent to the base of the articular eminence [8].

The articular eminence divergence angle was the angle measured between two lines: AE (anterior eminence line) and PE (posterior eminence line) in the obtained sagittal views [8].

The AE line (anterior eminence line) was traced as the best fitting line, which was tangent to the anterior wall of the articular eminence [8].

The PE line (posterior eminence line) was traced as the best fitting line, which was tangent to the posterior wall of the articular eminence [8].

**Figure 4:**
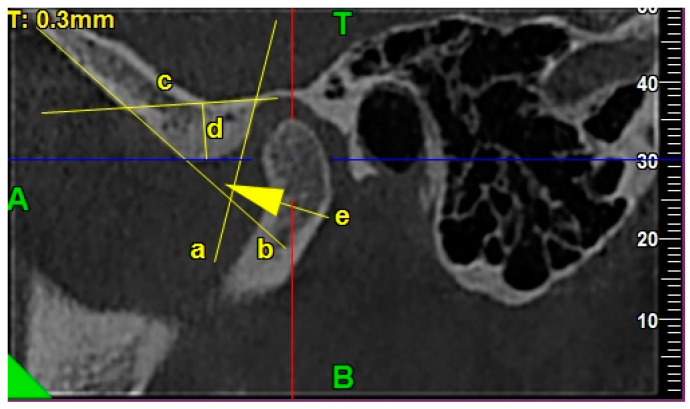
The exemplary lines, points, and angles in the TMJ CBCT scans used for measurements regarding the morphology of the articular eminence in the sagittal view: /a/ PE line, /b/ AE line, /c/ articular eminence basal line, /d/ articular eminence height, and /e/ articular eminence divergence angle.

Figure 4 presents the exemplary lines, points, and angles in the TMJ CBCT scans used for measurements regarding the morphology of the articular eminence in the sagittal view.

#### 2.4.4. Assessment of the Anterior, Posterior, and Superior Joint Spaces

Joint spaces were measured in the obtained sagittal view [8,11]. From the highest point of glenoid fossa, two lines were traced including one that approached the most posterior point of the condyle (CP-line), whereas the latter reached the most anterior point of the condyle (CA-line) [8]. 

Anterior joint space was perpendicular to the CA-line distance measured from the most anterior point of the condyle to the glenoid fossa [8].

Posterior joint space was perpendicular to CP-line distance measured from the most posterior point of the condyle to the glenoid fossa [8]. 

The superior joint space was a distance measured from the most superior point of glenoid fossa to the most superior point on the condylar head [8].

**Figure 5:**
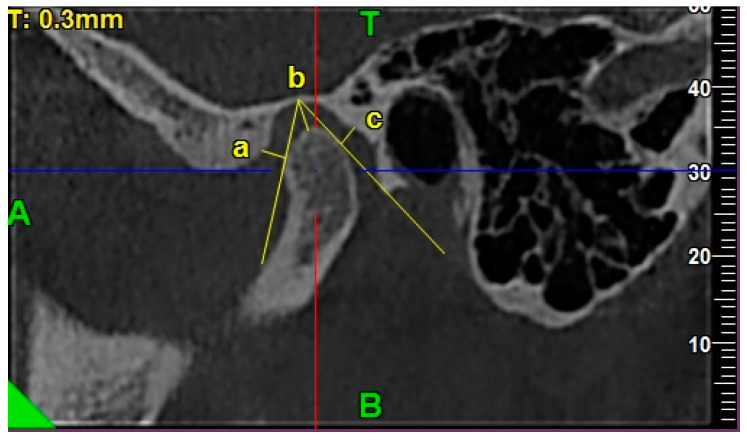
The exemplary lines, points, and angles in the TMJ CBCT scans used for measurements regarding the assessment of anterior, posterior, and superior joint spaces in the sagittal view: /a/ anterior joint space, /b/ superior joint space, and /c/ posterior joint space.

Figure 5 presents the exemplary lines, points, and angles in the TMJ CBCT scans used for measurements regarding the assessment of anterior, posterior, and superior joint spaces in the sagittal view.

#### 2.4.5. Assessment of the Sagittal Position of the Condyle 

The sagittal view of the condyle was assessed according to Pullinger and Hollender’s formula [12].

(1) $$condylar\;ratio=\frac{P-A}{P+A}x100\%\;$$

where:

*P*—posterior joint space,
*A*—anterior joint space.
The concentric position of the condyle was diagnosed if the condylar ratio was ±12%.
The posterior position of the condyle was diagnosed if the condylar ratio was <−12%.
The anterior position of the condyle was diagnosed if the condylar ratio was >12%.

#### 2.4.6. Frequency of the Osteoarthritic Osseous Changes 

**Figure 6:**
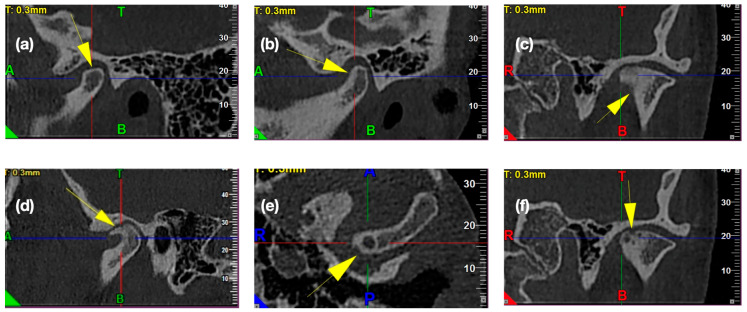
Osteoarthritic changes in the temporomandibular joints’ CBCT images: (**a**) Articular surface flattening, (**b**) subcortical sclerosis, (**c**) generalized sclerosis, (**d**) osteophyte, (**e**) subcortical cyst, and (**f**) erosion.

Temporomandibular joint osteoarthritis is diagnosed with the presence of below mentioned osseous changes [13]: flattening of the convex condylar head, erosion (the area of reduced density within the cortical layer and subcortical bone), osteophytes (osseous outgrowth on the surface of the mandibular condyle), sclerosis (increased density of dense cortical bone or increased density of bone tissue under the dense cortical bone), and pseudocyst (an osteolytic, well delimited, localized in the subcortical area, there is no destruction of the cortical layer of the bone in its course). The CBCT images were examined thoroughly to find the previously mentioned osseous changes. Figure 6 presents the exemplary osteoarthritic changes found in the TMJ CBCT scans. 

#### 2.4.7. Statistical Analysis 

To perform statistical analysis the StatSoft.Inc. (2014) STATISTICA (data analysis software system) version 12.0. was used. We measured: the mean value, standard deviation, median, minimum value, maximum value, and 95%CI (confidence interval). The below mentioned tests were used for statistical analysis: the Shapiro-Wilk, the Brown-Fosythe, T-Student test, Welch test, U Mann-Whitney, test F (ANOVA), Kruskal-Wallis test, the Chi-square test. To assess the correlation between two variables the Pearson correlation coefficient and/or Spearman’s rank correlation coefficient were measured. The statistical significance level was *p* = 0.05 for all the measurements included. 

## 3. Results

A total of 210 temporomandibular joints from 105 patients were analyzed. Table 1 presents the general characteristics of the examined patients. There were 25 patients with unilateral reciprocal clicking in TMJs, 25 patients with bilateral reciprocal clicking in TMJs, and 55 patients with no reciprocal clicking in TMJs. The percentage of women in groups with unilateral, bilateral, and with no TMJs’ clicks (control group) were: 68.0% vs. 84.0% vs. 74.5%, respectively. There were no statistically significant differences among the groups regarding sex (*p* = 0.4174).

The average age of the patients with unilateral TMJs’ clicks was 26.9 (9.0) years (range: 16–47 years), with bilateral TMJs’ clicks that were 23.0 (7.4) years (range: 16–42 years), and, for the control group with no TMJs’ clicks, the average age was 24.9 (7.3) years (range: 16–44 years). There were no statistically significant differences among the groups regarding age (*p* = 0.1545). 

The comparable characteristics of the different parameters related to condyle’s head, glenoid fossa, articular eminence, condylar head position in the glenoid fossa, and osteoarthritic changes in the area of the condylar head were performed between two groups: Group A (control group)—temporomandibular joints without reciprocal clicking (*N* = 135) and Group B—temporomandibular joints with reciprocal clicking (*N* = 75). 

Table 2 presents comparable characteristics of condyle’s head, glenoid fossa, and articular eminence as well as condylar head position in the glenoid fossa and osteoarthritic changes in the area of condylar head regarding the presence of reciprocal clicking.

There were no statistically significant differences regarding the distribution of the condyle head’s shapes between the examined groups (*p* = 0.2839).

The average condylar width in the control group (with no TMJs clicks) was 19.0 (2.1) mm (range: 13.4–23.9 mm), whereas, in the group with TMJs, reciprocal clicking was 18.7 (2.3) mm (range: 11.7–23.1 mm). There were no statistically significant differences regarding the condylar width between the examined groups (*p* = 0.4883). 

The average condylar A-P dimension in the control group (with no TMJs clicks) was 6.8 (1.2) mm (range: 3.5–11.7 mm), whereas, in the group with TMJs, reciprocal clicking was 6.3 (1.6) mm (range: 2.3–11.4 mm). The A-P dimension of the condylar head was significantly larger in the control group (with no reciprocal clicking) (*p* = 0.0399). 

**Figure 7:**
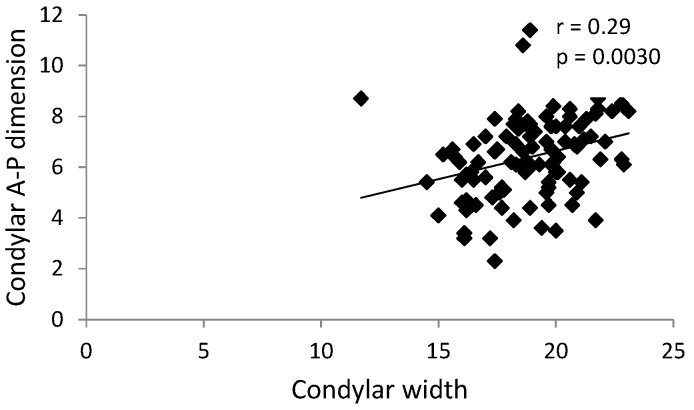
Graph presenting statistically significant positive correlation between the condylar A-P dimension and condylar width in the group with TMJ reciprocal clicking.

There was a statistically significant positive correlation (r = 0.29, p = 0.0030) between the condylar A-P dimension and condylar width in the group with reciprocal clicking (Figure 7). Such a correlation was not observed in the control group (without reciprocal clicking).

There were no statistically significant differences regarding the distribution of glenoid fossa’s shapes between the examined groups (*p* = 0.1261).

The average depth of glenoid fossa in the control group (with no TMJs clicks) was 9.8 (1.3) mm (range: 6.3–12.4 mm), whereas in the group with TMJs, reciprocal clicking was 9.7 (1.5) mm (range: 6.6–12.9 mm). There were no statistically significant differences regarding the depth of glenoid fossa between the examined groups (*p* = 0.5284).

The average length of glenoid fossa in the control group (with no TMJs clicks) was 20.5 (2.3) mm (range: 16.1–28.3 mm), whereas, in the group with TMJs, reciprocal clicking was 20.1 (1.9) mm (range: 15.6–26.4 mm). There were no statistically significant differences regarding the length of glenoid fossa between the examined groups (*p* = 0.3283).

The average divergence angle of glenoid fossa in the control group (with no TMJs clicks) was 56.7° (14.8°) (range: 26.0–100.5°), whereas, in the group with TMJs, reciprocal clicking was 55.3° (13.2°) (range: 29.2–94.2°). There were no statistically significant differences regarding the divergence angle of glenoid fossa between the examined groups (*p* = 0.7879).

**Figure 8:**
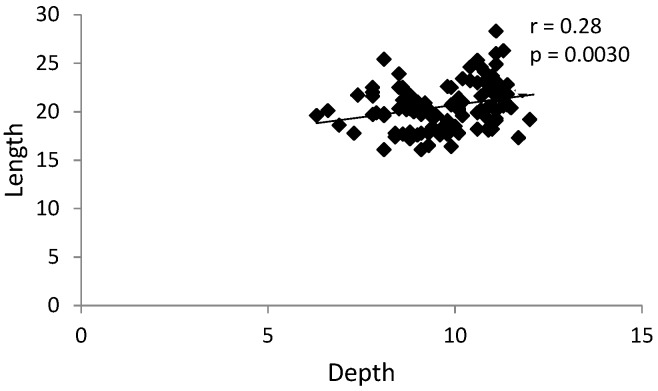
Graph presenting statistically significant positive correlation between the glenoid fossa depth and length in the control group (without TMJ reciprocal clicking).

**Figure 9:**
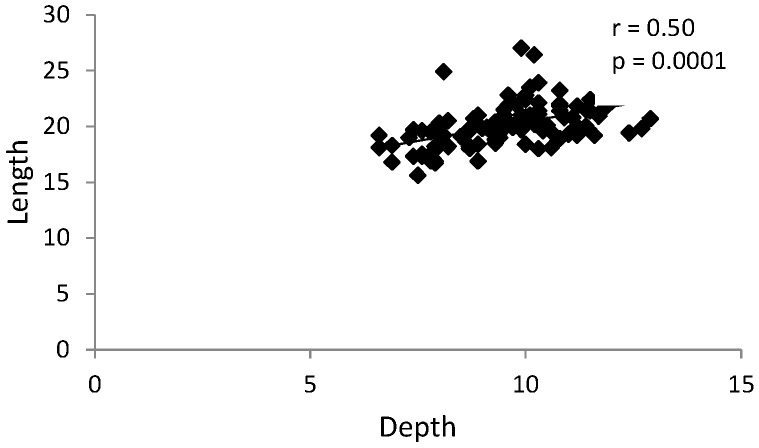
Graph presenting statistically significant positive correlation between the glenoid fossa depth and length in the group with TMJ reciprocal clicking.

There was a statistically significant positive correlation between the glenoid fossa depth and length in both groups (Figure 8 and Figure 9).

The average height of articular eminence in the control group (with no TMJs clicks) was 8.1 (2.1) mm (range: 3.6–13.4 mm), whereas, in the group with TMJs, reciprocal clicking was 8.0 (2.0) mm (range: 3.0–13.7 mm). There were no statistically significant differences regarding the height of articular eminence between the examined groups (*p* = 0.7227). 

The average divergence angle of articular eminence in the control group (with no TMJs clicks) was 82.9° (14.7°) (range: 49.7–127.6°), whereas, in the group with TMJs, reciprocal clicking was 81.9° (15.6°) (range: 43.7–115.1°). There were no statistically significant differences regarding the divergence angle of articular eminence between the examined groups (*p* = 0.6435).

The Condylar ratio, according to Pullinger and Hollender’s formula, was significantly lower in the group with TMJs reciprocal clicking −10.9% (23.2%) when compared to the control group (with no clicks) −2.9% (22.2%) (*p* = 0.0149). 

The distribution of the sagittal position of the condyle was significantly different in the group with reciprocal clicking when compared to the control group (*p* = 0.0425). In the group with reciprocal clicking, the condyle was positioned significantly more often in a posterior position and significantly less often in the anterior position.

**Figure 10:**
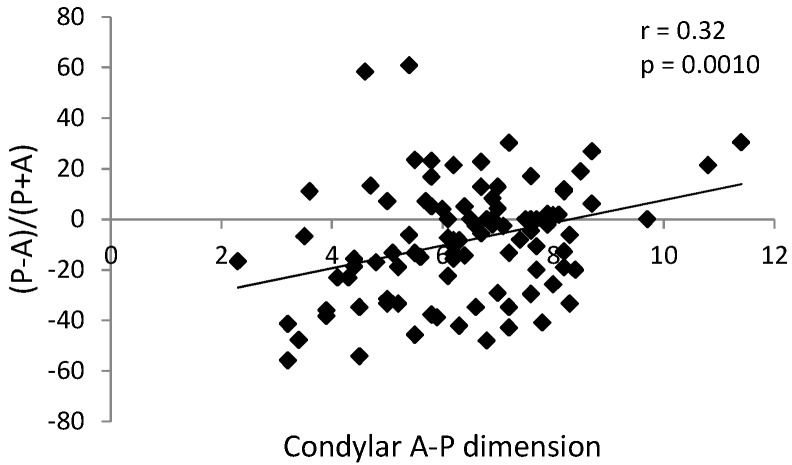
Graph presenting statistically significant positive correlation between condylar anteroposterior dimension and sagittal position of the condyle in the glenoid fossa in the group with TMJ reciprocal clicking.

**Figure 11:**
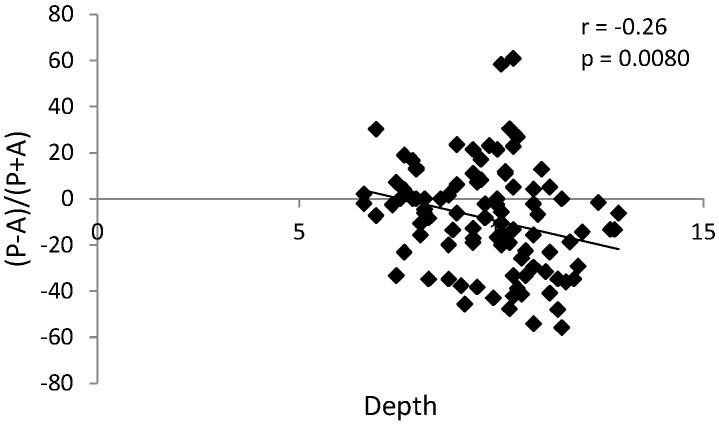
Graph presenting statistically significant negative correlation between glenoid fossa depth and sagittal position of the condyle in the glenoid fossa in the group with TMJ reciprocal clicking.

There were statically significant correlations in the TMJ reciprocal clicking group: positive (r = 0.32, *p* = 0.0010) between condylar anteroposterior dimension and sagittal position of the condyle in the glenoid fossa (Figure 10), and negative (r = −0.26, *p* = 0.0080) between glenoid fossa depth and sagittal position of the condyle in the glenoid fossa (Figure 11). These correlations were not observed in the control group (without TMJ reciprocal clicking).

**Figure 12:**
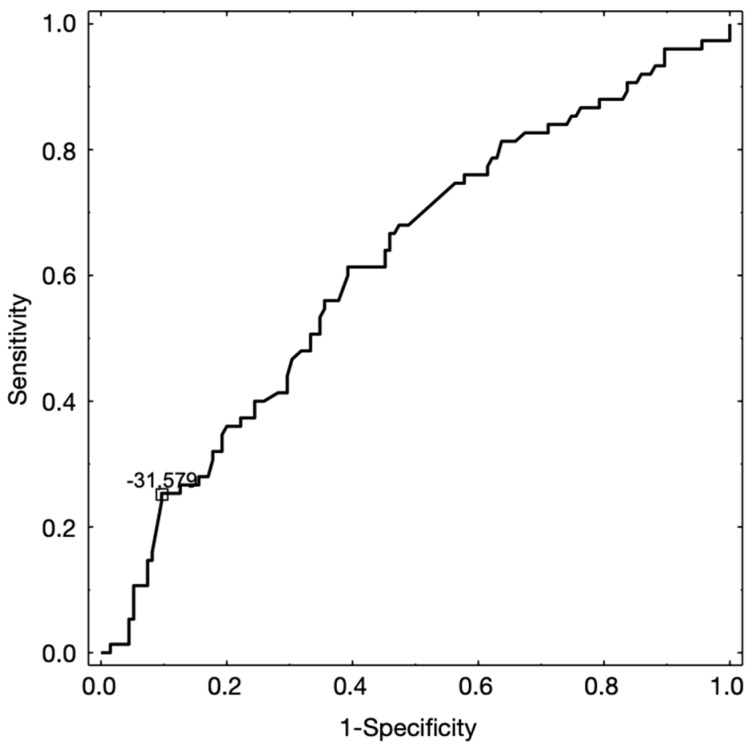
Diagram for receiver operating curve (ROC) analysis to assess the cut-off value of the parameter (P − A) / (P + A) between the groups with and without TMJ reciprocal clicking.

To assess the probability of the presence of the reciprocal clicking regarding the sagittal position of the condyle in the glenoid fossa, the ROC analysis was performed. The value of (P − A) / (P + A) = −31.579 was the cut-off point, which distinguished the reciprocal clicking and lack of it. The (P − A) / (P + A) with the value below −31.579 represented reciprocal clicking. The measured cut-off point was characterized by the sensitivity of 25.3%, and specificity of 90.4%. The value of area under the curve was: AUC = 0.62 (95%CI [0.54;0.70]), *p* = 0.0034). Figure 12 presents the diagram for receiver operating curve (ROC) analysis.

The subcortical cysts were diagnosed to be statistically significant more often in the group with reciprocal clicking when compared to the control group (*p* = 0.0003). There were no other significant differences between the previously mentioned groups concerning the remaining osteoarthritic changes. 

## 4. Discussion

Anterior disc displacement with reduction (ADDR) is the most common type of temporomandibular joint internal derangement. Its frequency among adults achieves the level of up to 35% [7]. ADDR has also been confirmed among young people with the peak during the years of adolescence (14.4% of teenagers aged: 13–18 years) [14]. Therefore, ADDR ought to be of special interest to orthodontists who most often treat teenagers and young adults. 

One of the typical clinical signs of ADDR is reciprocal clicking. Therefore, most of the practitioners rely on clinical examination in everyday practice. Though it may seem not enough to diagnose ADDR only based on the temporomandibular joints’ reciprocal clicking [7], some authors recommend relying primarily on the functional diagnostic approach [15], whereas, to increase the overall accuracy to 90%, others suggest a combination of the common clicking test with either the elimination test or, based on Dawson’s technique, the manipulation test [16]. If the condition of ADDR persists, further stages of internal derangements may be developed and, at the same time, may be accompanied by the osseous changes.

The most common shape of the condyle head in the coronal images in both groups was convex. Even though the percentage of other shapes’ distribution between the groups was noticeable, the differences were not significant. The predominance of the convex shape of the condyle head was also confirmed by de Farias et al. [17], Yale et al. [4], Ejima et al. [18], and Kijima et al. [19]. Unlike the previously mentioned authors, Santos et al. [20] most commonly diagnosed the flattened shape of the condyle head, which, according to the authors, indicated the initial phase of bone changes in the course of internal derangements. However, Santos et al. also included cases with anterior disc displacement without reduction (ADDwoR), which is a further stage of internal derangement when compared to ADDR. Patients with anterior disc displacement without reduction were excluded from our study. Therefore, the percentage of flattened condyles in our study may be reduced. Interestingly, Poluha et al. [21] recognized only two different condyles’ shapes: round (63.8%) and angled (36.2%). This result could likely have been the consequence of a small number of participants (36 people) included in the study. 

Condylar morphologies have been discussed by many authors, but most often only based on the condylar shape [17,19,21]. This study also analyzed the morphology of the condyle quantitatively by measuring the condylar width and condylar A-P dimension. There was no statistically significant difference in condylar width between the examined groups. However, the condylar A-P dimension was significantly smaller (*p* = 0.0399) in the group of TMJs with reciprocal clicking. Yasa et al. [11] noticed that both the mediolateral and anteroposterior widths of the condyle were significantly smaller in the TMJ dysfunction group when compared to the asymptomatic group. Seo et al. [22] found that condylar depth (A-P dimension) was significantly smaller in condyles with disc displacement without reduction (DDNR) when compared to joints with normal disc position (NR) or disc displacement with reduction (DDR). However, there was no significant difference between NR and DDR. They also found that the condylar width was significantly smaller in DDR compared to NR as well as in DDNR compared to DDR. Even though the significant differences differ among our studies, the general tendencies in measurements’ values remained the same. The average values of both the condyle width and depth were smaller in internal derangements groups, when compared to healthy TMJs control groups. The differences between the studies might have been the result of diverse reference points considered for measurements, and the number of participants included in the research.

No significant differences in morphology of glenoid fossa (shape, depth, length, and divergence angle) between both of the TMJs groups with and without reciprocal clicking were found. Achieved outcomes follow the results published by Poluha et al. [21], Sato et al. [23], and Almasan et al. [24].

This research also presents no statistically significant differences between the examined groups regarding the height and the divergence angle of the articular eminence. Hirata et al. [25] did not find any association between disc displacement with reduction and the shape of the articular eminence. Furthermore, according to the studies by Poluha et al. [21] and Galante et al. [26], there is no relationship between the articular eminence angulation and symptoms of temporomandibular joint dysfunction. Otherwise, Bedran et Santos [27] presented results showing that disc displacement with reduction was significantly associated with disc deformity as well as with a change in the shape of the articular eminence. Though it has to be emphasized that the authors assessed the articular eminence qualitatively, they did not include any measurements that could objectively support their previously mentioned thesis. 

Condyle sagittal anteroposterior position in glenoid fossa has been assessed by many authors. It is generally believed that the more posterior the condyle position is, the higher the risk for disc displacement is. Lin et al. [28] confirmed that anterior disc displacement is correlated with pain (to a greater extent in cases without reduction) due to exposure and compression of retro-discal tissues. According to our research, in cases with reciprocal clicking, condyles were more often positioned posteriorly. There were no differences in the height of the superior joint space between groups. The average value of Pullinger and Hollender’s index was significantly lower in the group of TMJs with clicking. Yasa et al. [11] achieved similar results when considering superior and posterior joint space. They also noticed that anterior joint space was significantly larger in the group of TMJ dysfunction patients when compared to the asymptomatic group. Likewise, Ikeda et al. [29] recognized a significantly larger mean anterior space and significantly smaller mean posterior joint space in cases with disc displacement.

Contrary to our results, Ikeda et al. [29] noticed significantly smaller superior joint space in TMJs with total disc displacement with reduction. The probable explanation of this discrepancy might be the speculation that the majority of our “reciprocal clicking” patients could have had partially (not totally) displaced discs. According to the authors, superior joint space was similar in cases with normal sagittal disc position and partial disc displacement. Poluha et al. [21] did not confirm any differences in sizes of articular spaces among control and internal derangement groups nor found any association between the sizes of articular spaces and symptoms of TMJ arthralgia. In the authors’ opinion, patients’ adaptability and susceptibility to pain were responsible for the uncommon results. Rabelo et al. [30] found the association between anterior disc displacement and an increased size of anterior joint space. Almasan et al. [24] noticed that, in cases with anterior disc displacement with reduction compared to the group with the normal position of the disk, only anterior joint space was significantly larger. Moreover, they did not confirm any significant differences concerning the superior and posterior joint spaces between the previously mentioned groups. Even though the differences were not significant, the tendency to decrease the posterior joint space in cases with anterior disc displacement with reduction was noticeable. The reason behind the achieved results could have been the small amount of examined group. The research by Almasan et al. [24] was based on 34 participants, including 27 joints with disc displacement with reduction, 16 joints with disc displacement without reduction, and 31 joints with correctly positioned discs. 

Osteoarthritis is considered to be the joint disease that occurs most frequently. It is more common in females than in males. In the course of osteoarthritis, not only does the articular cartilage become degraded, but also the whole joint is affected, including subchondral bone and synovium [31]. According to our research, osteoarthritic osseous changes were diagnosed in both groups. Articular surface flattening was the most common osseous change in both groups with and without reciprocal clicking, but no significant differences between them were discovered. Only subcortical cysts occurred significantly more often in the group of TMJs with clicking. Conversely, other authors had different observations in the field of osteoarthritic changes. Dias et al. [32] found a statistically significant correlation between anterior disc displacement with reduction and condylar flattening. They described more correlations between degenerative TMJ changes and anterior disc displacement without reduction, which is not the issue of our research. De Melo et al. [33] did not observe any significant correlation regarding the anterior disc displacement with reduction and osseous changes. Nonetheless, they noticed a statistically significant correlation between bilateral disk displacement without reduction and pain and osseous changes. The differences among our outcomes could have been the consequences of different methodologies. The authors assessed osseous changes in magnetic resonance images (MRI). MRI is a “gold standard” for soft tissue examination, including articular disc morphology and position, but for osseous changes, diagnosis computed tomography or cone-beam computed tomography is a preferable method of imaging.

Some authors proved that internal derangements might increase the risk of osteoarthritis [34,35]. Roh et al. [34] stated that the joints with anterior disk displacement with reduction showed a 2.01 odds ratio of degenerative changes, and, in cases without reduction, the odds ratio of degenerative changes increased up to 4.43. Dias et al. [35] presented even greater odds ratios relating to respective internal derangements. According to their study, temporomandibular joints with anterior disc displacement with reduction appeared to develop osteoarthrosis 2.73-times more likely, whereas joints with anterior disc displacement without reduction appeared to cause osteo-arthrosis 8.25-times more likely. The authors claimed that both the internal derangements and degenerative osseous changes are closely related to each other.

This study has some potential limitations. First of all, the examined subjects were referred for orthodontic consultation because of the presence of malocclusion. This could have altered the prevalence of TMJ morphological alterations when compared to a normal population. Second, the diagnosis of the disc displacement was based only on clinical examination. Although, some of the authors recommend that clinical examination is enough to diagnose disc displacement. It has to be emphasized that a method of choice to diagnose the morphology and position of the articular disc is magnetic resonance imaging. Third, the range of age of examined was from 16 to 47 years old. It would be valuable to include elderly people in further studies.

## 5. Conclusions

Temporomandibular joints with reciprocal clicking were characterized by significantly smaller condylar A-P dimension. Condyles were significantly more often positioned posteriorly and significantly more often were accompanied by subcortical cysts, pathologic osteoarthritic bone changes. It is highly recommended to examine temporomandibular joints thoroughly before each type of dental treatment with a special consideration in orthodontics and prosthodontics. TMJs radiological examination gives additional information to functional diagnostics and is often necessary in treatment planning.

## Figures and Tables

**Table 1:** General characteristics (including sex and age) of patients with unilateral, bilateral, and without any clicks in temporomandibular joints (TMJs).

Comparable Characteristics	Unilateral Clicks (*N* = 25)	Bilateral Clicks (*N* = 25)	Control (No Clicks) (*N* = 55)	*p*-Value
**Age**				0.1545 ^1^
av. (SD)	26.9 (9.0)	23.0 (7.4)	24.9 (7.3)	
range	16–47	16–42	16–44	
median	24.8	19.2	22.7	
95% CI	[23.2; 30.6]	[20.0; 26.1]	[22.9;26.8]	
**Sex**				0.4174 ^2^
female	17 (68.0%)	21 (84.0%)	41 (74.5%)	
male	8 (32.0%)	4 (16.0%)	14 (25.5%)	

^1^ Kruskal-Wallis, ^2^ Chi-square.

**Table 2:** Comparable characteristics of condyle’s head, glenoid fossa, and articular eminence as well as condylar head position in glenoid fossa and osteoarthritic changes in the area of condylar head regarding the presence of reciprocal clicking.

Comparable Characteristics	Control Group (TMJs with no Clicks)	TMJ’s with Reciprocal Clicking	*p*-Value
	(*N* = 135)	(*N* = 75)	
CONDYLE			
**Shape of condyle head**			0.2839 ^1^
flattened	30 (22.2%)	19 (25.3%)	
convex	57 (42.2%)	23 (30.7%)	
angled	18 (13.4%)	16 (21.3%)	
round	30 (22.2%)	17 (22.7%)	
**Condylar width [mm]**			0.4883 ^2^
av. (SD)	19.0 (2.1)	18.7 (2.3)	
range	13.4–23.9	11.7–23.1	
median	19.2	18.8	
25–74% percentile	17.4–20.4	17.2–20.4	
95% CI	[18.6;19.3]	[18.2;19.3]	
**Condylar A-P dimension [mm]**			0.0399 ^3^
av. (SD)	6.8 (1.2)	6.3 (1.6)	
range	3.5–11.7	2.3–11.4	
median	6.7	6.3	
25–74% percentile	6.0–7.5	5.4–7.4	
95% CI	[6.6;7.0]	[5.9;6.6]	
**GLENOID FOSSA**			
**Shape**			0.1261 ^1^
oval	77 (57.0%)	53 (70.7%)	
trapezoidal	31 (23.0%)	15 (20.0%)	
triangular	18 (13.3%)	6 (8.0%)	
angled	9 (6.7%)	1 (1.3%)	
**Depth [mm]**			0.5284 ^3^
av. (SD)	9.8 (1.3)	9.7 (1.5)	
range	6.3–12.4	6.6–12.9	
median	9.9	9.9	
25–74% percentile	8.7–10.9	8.7–10.5	
95% CI	[9.5; 10.0]	[9.3; 10.0]	
**Length [mm]**			0.3283 ^3^
av. (SD)	20.5 (2.3)	20.1 (1.9)	
range	16.1–28.3	15.6–26.4	
median	20.2	20.1	
25–74% percentile	19.1–21.7	19.1–21.4	
95% CI	[20.1; 20.9]	[19.7; 20.6]	
**Divergence angle [°**]			0.7879 ^3^
av. (SD)	56.7 (14.8)	55.3 (13.2)	
range	26.0–100.5	29.2–94.2	
median	55.0	56.4	
25–74% percentile	44.8–64.7	44.5–64.6	
95% CI	[54.2; 59.3]	[52.3; 58.4]	
**ARTICULAR EMINENCE**			
**Height [mm]**			0.7227 ^2^
av. (SD)	8.1 (2.1)	8.0 (2.0)	
range	3.6–13.4	3.0–13.7	
median	8.2	8.1	
25–74% percentile	6.6–9.6	6.6–9.3	
95% CI	[7.8; 8.5]	[7.5; 8.5]	
**Divergence angle [°**]			0.6435 ^2^
av. (SD)	82.9 (14.7)	81.9 (15.6)	
range	49.7–127.6	43.7–115.1	
median	81.0	83.2	
25–74% percentile	73.0–90.8	69.8–91.4	
95% CI	[80.4; 85.4]	[78.3; 85.5]	
**CONDYLAR HEAD POSITION**			
**(P-A)/(P+A) [%]**			0.0149 ^2^
av. (SD)	−2.9 (22.2)	−10.9 (23.2)	
range	−69.4–56.0	−55.8–60.9	
median	0.0	−10.5	
25–74% percentile	−15.8–11.6	−31.6–2.0	
95% CI	[−6.7;0.9]	[−16.2; −5.6]	
**Superior**			0.8566 ^2^
av. (SD)	3.2 (0.9)	3.2 (1.0)	
range	1.2–6.1	1.2–6.5	
median	3.3	3.1	
25–74% percentile	2.7–3.8	2.5–3.6	
95% CI	[3.1;3.4]	[3.0;3.4]	
**Position**			0.0425 ^1^
anterior	32 (23.7%)	10 (13.3%)	
posterior	43 (31.9%)	36 (48.0%)	
concentric	60 (44.4%)	29 (38.7%)	
**OSTEOARTHRITIC CHANGES**			
**Number of osteoarthritic changes**			0.0152 ^3^
av. (SD)	2.3 (1.2)	1.8 (1.1)	
range	0.0–5.0	0.0–5.0	
median	2	2	
25–74% percentile	1.0–3.0	1.0–2.0	
95% CI	[2.0; 2.5]	[1.6; 2.1]	
**Subcortical sclerosis**	39 (28.9%)	31 (41.3%)	0.0668 ^1^
**Osteophyte**	32 (23.7%)	25 (33.3%)	0.1327 ^1^
**Subcortical cyst**	7 (5.2%)	16 (21.3%)	0.0003 ^1^
**Surface erosion**	55 (40.7%)	33 (44.0%)	0.6465 ^1^
**Articular surface flattening**	121 (89.6%)	68 (90.7%)	0.8103 ^1^
**Generalized sclerosis**	0 (0.0%)	2 (2.7%)	0.0566 ^1^

^1^ Chi-square, ^2^ t-Student, ^3^ U Mann-Whitney, statistically significant *p*-values are written in red.

## References

[1] Okeson J.P. (2020). Management of Temporomandibular Disorders and Occlusion.

[2] Ottria L., Candotto V., Guzzo F., Gargari M., Barlattani A. (2018). Temporomandibular joint and related structures: Anatomical and Histological aspects. J. Biol. Regul. Homeost. Agents.

[3] Stocum D.L., Roberts W.E. (2018). Part I: Development and physiology of the temporomandibular joint. Curr. Osteoporos Rep..

[4] Yale S.H., Allison B.D., Hauptfuehrer J.D. (1966). An epidemiological assessment of mandibular condyle morphology. Oral Surg. Oral Med. Oral Pathol..

[5] Ahmad M., Schiffman E.L. (2016). Temporomandibular joint disorders and orofacial pain. Dent. Clin. North. Am..

[6] Huddleston Slater J.J.R., Onland-Moret N.C., Lobbezoo F., Naeije M. (2007). Anterior disc displacement with reduction and symptomatic hypermobility in the human temporomandibular joint: Prevalence rates and risk factors in children and teenagers. J. Orofac. Pain.

[7] Naeije M., Te Veldhuis A.H., Te Veldhuis E.C., Visscher C.M., Lobbezoo F. (2013). Disc displacement within the human temporomandibular joint: A systematic review of a ‘noisy annoyance’. J. Oral Rehabil..

[8] Derwich M., Mitus-Kenig M., Pawlowska E. (2020). Morphology of the temporomandibular joints regarding the presence of osteoarthritic changes. Int. J. Environ. Res. Public Health.

[9] Ikeda K. (2014). TMJ 1st Orthodontics Concepts, Mechanics, and Stability.

[10] Nguyen T., Proffit W., Graber L.W., Vanarsdall R.L., Vig K.W.L., Huang G.J. (2017). The decision-making process in orthodontics. Orthodontics Current Principles and Techniques.

[11] Yasa Y., Akgul H.M. (2018). Comparative cone-beam computed tomography evaluation of the osseous morphology of the temporomandibular joint in temporomandibular dysfunction patients and asymptomatic individuals. Oral Radiol..

[12] Pullinger A.G., Hollender L., Solberg W.K., Petersson A. (1985). A tomographic study of mandibular condyle position in an asymptomatic population. J. Prosthet. Dent..

[13] Ahmad M., Hollender L., Anderon Q., Kartha K., Ohrbach R., Truelove E.L., John M.T., Schiffman E.L. (2009). Research diagnostic criteria for temporomandibular disorders (RDC/TMD): Development of image analysis criteria and examiner reliability for image analysis. Oral Surg. Oral Med. Oral Pathol. Oral Radiol. Endod..

[14] Marpaung C., van Selms M.K.A., Lobbezoo F. (2019). Temporomandibular joint anterior disc displacement with reduction in a young population: Prevalence and risk indicators. Int. J. Paediatr. Dent..

[15] Marpaung C.M., Kalaykova S.I., Lobbezoo F., Naeije M. (2014). Validity of functional diagnostic examination for temporomandibular joint disc displacement with reduction. J. Oral Rehabil..

[16] Yatani H., Sonoyama W., Kuboki T., Matsuka Y., Orsini M.G., Yamashita A. (1998). The validity of clinical examination for diagnosing anterior disk displacement with reduction. Oral Surg. Oral Med. Oral Pathol. Oral Radiol. Endod..

[17] de Farias J.F., Melo S.L.S., Bento P.M., Oliveira L.S.A.F., Campos P.S.F., de Melo D.P. (2015). Correlation between temporomandibular joint morphology and disc displacement by MRI. Dentomaxillofac. Radiol..

[18] Ejima K., Schulze D., Stippig A., Matsumoto K., Rottke D., Honda K. (2013). Relationship between the thickness of the roof of glenoid fossa, condyle morphology and remaining teeth in asymptomatic European patients based on cone beam CT data sets. Dentomaxillofac. Radiol..

[19] Kijima N., Honda K., Kuroki Y., Sakabe J., Ejmia K., Nakajima I. (2007). Relationship between patient characteristics, mandibular head morphology and thickness of the roof of the glenoid fossa in symptomatic temporomandibular joints. Dentomaxillofac. Radiol..

[20] Santos K.C., Dutra M.E., Warmling L.V., Oliveira J.X. (2013). Correlation among the changes observed in temporomandibular joint internal derangements assessed by magnetic resonance in symptomatic patients. J. Oral. Maxillofac. Surg..

[21] Poluha R.L., Cunha C.O., Bonjardim L.R., Conti P.C.R. (2020). Temporomandibular joint morphology does not influence the presence of arthralgia in patients with disk displacement with reduction: A magnetic resonance imaging-based study. Oral Surg. Oral Med. Oral Pathol. Oral Radiol..

[22] Seo B.Y., An J.S., Chang M.S., Huh K.H., Ahn S.J. (2020). Changes in condylar dimensions in temporomandibular joints with disk displacement. Oral Surg. Oral Med. Oral Pathol. Oral Radiol..

[23] Sato S., Kawamura H., Motegi K., Takahashi K. (1996). Morphology of the mandibular fossa and the articular eminence in temporomandibular joints with anterior disk displacement. Int. J. Oral Maxillofac. Surg..

[24] Almasan O.C., Hedesiu M., Baciut G., Leucuta D.C., Baciut M. (2013). Disk and joint morphology variations on coronal and sagittal MRI in temporomandibular joint disorders. Clin. Oral Investig..

[25] Hirata F.H., Guimaraes A.S., de Oliveira J.X., Moreira C.R., Ferreira E.T.T., Cavalcanti M.G.P. (2007). Evaluation of TMJ articular eminence morphology and disc patterns in patients with disc displacement in MRI. Braz. Oral Res..

[26] Galante G., Paesani D., Tallents R.H., Hatala M.A., Katzberg R.W., Murphy W. (1995). Angle of the articular eminence in patients with temporomandibular joint dysfunction and asymptomatic volunteers. Oral Surg. Oral Med. Oral Pathol. Oral Radiol. Endod..

[27] Bedran L.M., Santos A.A.S.M.D. (2019). Changes in temporomandibular joint anatomy, changes in condylar translation, and their relationship with disc displacement: Magnetic resonance imaging study. Radiol. Bras..

[28] Lin W.C., Lo C.P., Chiang I.C., Hsu C.C., Hsu W.L., Liu D.W., Juan Y.H., Liu G.C. (2012). The use of pseudo-dynamic magnetic resonance imaging for evaluating the relationship between temporomandibular joint anterior disc displacement and joint pain. Int. J. Oral Maxillofac. Surg..

[29] Ikeda K., Kawamura A. (2013). Disc displacement and changes in condylar position. Dentomaxillofac. Radiol..

[30] Rabelo K.A., Sousa Melo S.L., Torres M.G.G., Peixoto L.R., Campos P.S.F., Rebello I.M.C.R., de Melo D.P. (2017). Assessment of condyle position, fossa morphology, and disk displacement in symptomatic patients. Oral Surg. Oral Med. Oral Pathol. Oral Radiol..

[31] Glyn-Jones S., Palmer A.J.R., Agricola R., Price A.J., Vincent T.L., Weinans H., Carr A.J. (2015). Osteoarthritis. Lancet.

[32] Dias I.M., Coelho P.R., Picorelli Assis N.M.S., Pereira Leite F.P., Devito K.L. (2012). Evaluation of the correlation between disc displacements and degenerative bone changes of the temporomandibular joint by means of magnetic resonance images. Int. J. Oral Maxillofac. Surg..

[33] de Melo D.P., Sousa Melo S.L., de Andrade Freitas Oliveira L.S., Ramos-Perez F.M., Campos P.S. (2015). Evaluation of temporomandibular joint disk displacement and its correlation with pain and osseous abnormalities in symptomatic young patients with magnetic resonance imaging. Oral Surg. Oral Med. Oral Pathol. Oral Radiol..

[34] Roh H.S., Kim W., Kim Y.K., Lee J.Y. (2012). Relationships between disk displacement, joint effusion, and degenerative changes of the TMJ in TMD patients based on MRI findings. J. Craniomaxillofac. Surg..

[35] Dias I.M., Cordeiro P.C., Devito K.L., Tavares M.L.F., Leite I.C.G., Tesch R. (2016). Evaluation of temporomandibular joint disc displacement as a risk factor for osteoarthrosis. Int. J. Oral Maxillofac. Surg..

